# Spike-timing-dependent plasticity steers new Parkinson-stimulation protocols

**DOI:** 10.1186/1471-2202-14-S1-P401

**Published:** 2013-07-08

**Authors:** Marcel AJ Lourens, Jasmine A Nirody, Hil GE Meijer, Lo J Bour, Stephan A van Gils

**Affiliations:** 1 Academic Medical Center, University of Amsterdam, Amsterdam, 1105 AZ, The Netherlands; 2 Department of Applied Mathematics, University of Twente, Enschede, 7500 AE, The Netherlands; 3 Biophysics Graduate Group, University of California, Berkeley, Berkeley, CA 94720, USA

## Introduction

In advanced Parkinson's disease (PD), deep brain stimulation (DBS) can be used to disrupt pathological activity in the basal ganglia, thereby reducing PD motor symptoms. The standard protocol for DBS, continuous high frequency stimulation of target cells, is applied notably in subthalamic nucleus (STN) or globus pallidus pars interna. It is proposed that short-duration desynchronizing stimulation protocols may also disrupt pathological synchronous activity [1]. Synaptic plasticity is supposed to be the underlying mechanism. The goal of this study is to explore, with a biophysically plausible model, the role of synaptic plasticity in stabilizing firing patterns in the basal ganglia. Moreover, we investigate how STN stimulation should be applied, such that it exploits synaptic plasticity most effectively to bring the network in a less synchronous state.

## Methods

We use an existing STN-GPe network model consisting of 16 STN and globus pallidus pars externa (GPe) cells mutually connected via a sparse structured architecture [2]. The dynamics of each cell is described by a single-compartment conductance-based model. We augment the model with a rule for spike-timing-dependent plasticity (STDP) for the inhibitory connections within GPe. The synaptic weight is updated with an additive nearest-spike pair-based STDP rule. We apply DBS as a train of positive current pulses (*P*(*t*)) injected directly into each STN cell. *P*(*t*) is admin¬istered according to two different protocols: 1) Standard DBS, i.e. continuous stimu¬lation, in which all STN cells receive exactly the same stimulation signal; 2) Coordinated Reset (CR) stimulation, in which the STN population is subdivided in 4 groups. Each STN group receives its own *P*(*t*) which is periodically on and off.

## Results and discussion

An STDP rule that down-/up-regulates the synaptic weights between GPe cells when they fire in synchronized/uncorrelated manner, stabilizes network states. Both a healthy state with desynchronized dynamics and a PD state with synchronized dynamics stably coexist. Depending on the original firing pattern, the network with STDP is 'learning' either pathological or healthy dynamics by adapting its pattern of the synaptic couplings between the GPe cells. Since DBS can change the firing pattern of the stimulated cells, it can be used to 'teach' the network with STDP to display more healthy activity. Our results suggest that when a travelling wave short-duration desynchronizing stimulation is applied sufficiently long and with sufficiently high amplitude, it may profit from STDP to train the network to fire in a less pathological manner. In contrast, STDP has a negative effect when continuous stimulation is employed, in the sense that the network becomes more synchronized when stimulation is switched off. Since with this kind of stimulation most of the time DBS is turned off, it saves battery power and it leads to fewer negative side effects of DBS in comparison to the traditional continuous high frequency stimulation.
